# Rapid Transit Again

**Published:** 1891-04

**Authors:** 


### RAPID TRANSIT AGAIN.

About a year ago we called attention to the advantages of the Safety bicycle for the use of physicians. Chief among these advantages we mentioned the rapidity with which their visits could be made. Since that time a Doctors' Wheel Club has been organized with over twenty members, and we doubt not that the present season will see that number considerably increased. It is not our intention at this time to repeat what has been said upon this subject—it is the practical every-day demonstration of seeing many physicians making their daily visits upon "the wheel." That is the best answer to the prejudice or credulity of those who think the bicycle is simply a thing for sport and not a vehicle for business purposes. We desire merely to direct those who are contemplating the purchase of a wheel to the notice of the machine called the "Swift," that appears upon one of our advertising pages. This wheel is built by the Coventry Machinist Company, of Coventry, England, one of the oldest of makers, and who are particularly famous for embodying in their wheels the principles of simplicity and strength. The "Swift" easily ranks with the first of the high grade safeties, and has the reputation of standing an immense amount of hard usage without any part giving way. These wheels are built in various weights and sizes, thereby recognizing that all men are not turned out of the same mold—the short, light-weight man not requiring the same amount of weight that the large, heavy man needs. We believe a wheel can be selected from among the "Swifts" that will meet all that is required by a physician in the use of a bicycle in his practice.

